# CCL21 activation of the MALAT1/SRSF1/mTOR axis underpins the development of gastric carcinoma

**DOI:** 10.1186/s12967-021-02806-5

**Published:** 2021-05-17

**Authors:** Qianmei Fu, Xiaohong Tan, Huaming Tang, Jijiang Liu

**Affiliations:** 1 Oncology Department, The People’s Hospital of Kaizhou District, Chongqing, 405400 People’s Republic of China; 2 General Surgery Department, The People’s Hospital of Kaizhou District, Chongqing, 405400 People’s Republic of China; 3 Department of Gastroenterology, The People’s Hospital of Kaizhou District, No. 8, Ankang Road, Kaizhou District, Chongqing, 405400 People’s Republic of China; 4 Department of Otorhinolaryngology, The People’s Hospital of Kaizhou District, No. 8, Ankang Road, Kaizhou District, Chongqing, 405400 People’s Republic of China

**Keywords:** C–C motif chemokine ligand 21, Metastasis-associated lung adenocarcinoma transcript-1, Serine arginine-rich splicing factor 1, Mammalian target of rapamycin, Gastric carcinoma

## Abstract

**Background:**

As a significant cause of malignancy mortality, gastric carcinoma (GC) has been well documented to be an often-fatal diagnosis. Despite the limitations of effective therapy, immunotherapy has emerged as a promising therapeutic approach capable of killing cancer cells via the immune system. The current study was conducted to investigate the effect of cytokine C–C motif chemokine ligand 21 (CCL21) on GC progression through the metastasis-associated lung adenocarcinoma transcript 1/serine arginine-rich splicing factor 1/mammalian target of rapamycin (MALAT1/SRSF1/mTOR) axis.

**Methods:**

Bioinformatics analysis was conducted to identify the key genes associated with GC and to subsequently predict their downstream genes. The effect of CCL21, MALAT1, and SRSF1 on the malignant phenotypes and epithelial-mesenchymal transition (EMT) of SGC-7901 and MGC-803 cells *in-vitro* and the tumorigenesis of SGC-7901 and MGC-803 cells *in-vivo* were assessed by expression determination and plasmid transfection. Additionally, RNA pull-down and RNA binding protein immunoprecipitation experiments were performed to determine the MALAT1-microRNA-202-3p (miR-203-3p) interaction and miR-202-3p-SRSF1 interaction followed by the analysis of their effect on the mTOR pathway.

**Results:**

CCL21 was identified as a key GC immune gene. Overexpressed CCL21, MALAT1, and SRSF1 along with poorly expressed miR-202-3p were identified in the GC cells. CCL21 induced the MALAT1 expression in a time- and dose-dependent manner. Functionally, MALAT1 targeted miR-202-3p but upregulated SRSF1 and activated mTOR. Crucially, evidence was obtained indicating that CCL21 promoted both the malignant phenotypes and EMT of SGC-7901 and MGC-803 cells *in-vitro* and the tumorigenesis of SGC-7901 and MGC-803 cells *in-vivo* by increasing the MALAT1-induced upregulation of SRSF1.

**Conclusions:**

Taken together, the key observations of our study provide evidence that CCL21 enhances the progression of GC via the MALAT1/SRSF1/mTOR axis, providing a novel therapeutic target for the treatment of GC.

**Supplementary Information:**

The online version contains supplementary material available at 10.1186/s12967-021-02806-5.

## Introduction

As a malignancy of the stomach, gastric carcinoma (GC) ranks the fifth most common cancer while representing the third primary cause of cancer-associated death worldwide, posing a significant threat to public health globally [1]. *Helicobacter pylori* infection, Epstein-Barr virus, and heritable pathogenic mutation are considered to be major risk factors associated with GC [2]. Aberrant activation of epithelial-mesenchymal transition (EMT) in the stomach has implicated in gastric carcinogenesis and cancer development [3]. However, the aberrant activation of gastric EMT has been reported to display more mesenchymal features and less epithelial characteristics in gastric epithelial cells with studies highlighting its role as a facilitator of cancer cell stemness, malignant phenotypes, and chemo-resistance. Moreover, this phenomenon has been demonstrated to exert an inhibitory effect on cellular adhesion molecules including E-cadherin, ultimately permitting the spread of cancer cells throughout the body [4]. Although the one-year survival rate of patients with GC has been reported to have increased to 49% from 1984 to 2013, the treatment for GC remains somewhat limited [5]. The major traditional therapeutic options for GC include surgery and radiotherapy; however, the results of these approaches are at times unsatisfactory [6]. Immunotherapy has emerged as promising therapy capable of harnessing the immune system to kill cancer cells. Additionally, the ability of cytokines to modify biological responses continues to be explored owing to its potential to activate anti-cancer immune responses [7].

Intriguingly, the data we obtained from the bioinformatics analysis suggested the cytokine C–C motif chemokine ligand 21 (CCL21), or secondary lymphoid chemokine as the key gene of GC. CCL21 is a member of the CC chemokines that has been reported to co-localize the dendritic cells and lymphocytes, emphasizing its promise as a therapeutic target for human solid cancers [8]. Moreover, CCL21 has been previously documented to be overexpressed in GC [9], highlighting its potential oncogenic role in this malignancy. However, to date, the potential mechanism by which CCL21 influences GC remains undefined. Thus, the central objective of the present study was to elucidate the role of CCL21 in connection to the tumorigenesis of GC. Existing literature has revealed that CCL21 upregulates the expression of metastasis-associated lung adenocarcinoma transcript 1 (MALAT1) [10]. The potential role of MALAT1 in various cancers has been speculated particularly in relation to liver and digestive system cancers with its overexpression also reported in GC [11]. Accumulating evidence has revealed that MALAT1 enhances the expression of serine arginine-rich splicing factor 1 (SRSF1) and activates the mammalian target of rapamycin (mTOR) signaling pathway in hepatocellular carcinoma (HCC). Intriguingly, upregulated SRSF1 and activated mTOR plays a crucial role in tumorigenesis [12]. As a member of SR family that has reported to be upregulated in many cancer types including GC, SRSF1 is regarded as a novel biomarker for GC [13, 14]. Furthermore, activation of the mTOR pathway promotes cell progression and is responsible for dismal prognosis in GC [15]. Additionally, the mTOR pathway has been indicated to regulate the EMT process [16]. Based on these aforementioned findings, we asserted the hypothesis that CCL21 could mediate the EMT via the MALAT1/SRSF1/mTOR axis to promote the development of GC.

## Methods

### Ethics statement

All study protocols were performed with the approval of the Ethics Committee of The People’s Hospital of Kaizhou District and conducted in strict adherence with the Declaration of Helsinki. All patients and/or legal guardians signed informed consent documentation prior to enrollment into the study. All animal experiments were approved by the Animal Ethics Committee of The People’s Hospital of Kaizhou District. Extensive efforts were made to minimize the suffering and number of animals during the study.

### Bioinformatics analysis

The human immune genes were obtained from ImmPort (https://www.immport.org/home). Following differential analysis of stomach adenocarcinoma (STAD) dataset in The Cancer Genome Atlas (TCGA) using the R package limma (http://www.bioconductor.org/packages/release/bioc/html/limma.html), GC-related differentially expressed immune genes (∣logFoldChange∣ > 1, *p* < 0.05) were screened followed by single-factor Cox analysis with GC clinical data in the TGCA (http://www.bioconductor.org/packages/release/bioc/html/limma.html) using “survival” package (https://cran.r-project.org/web/packages/survival/index.html) to identify the genes associated with GC prognosis [17]. The hazard ratio (HR) and *p* value of each differential immune gene with the survival of GC patients were calculated, and the immune genes significantly related to the prognosis of GC patients were screened out based on the criteria of *p* < 0.05. Survival-related genes in the multivariate Cox regression analysis were inferred using the least absolutes shrinkage and selection operator (LASSO) by the R package glmnet. Risk scores were obtained according to genes expression multiplied by a linear combination of regression coefficient acquired from the multivariate Cox regression. Subsequently, according to the risk scores, the patients were classified into 158 high-risk cases and 159 low-risk cases based on the optimal cut-off point of risk score using the R package survminer. A survival curve was then constructed via survival analysis to determine the difference in terms of survival between high-risk cases and low-risk cases, while its credibility was confirmed by plotting the relative operating characteristic (ROC) curve. Through comparing differential expression of the genes involved in the GC prognostic risk model, the most significantly differentially expressed genes were subsequently selected as the key genes. The downstream pathways of the key genes were then identified based on previous literature. LncBase (http://carolina.imis.athena-innovation.gr/diana_tools/web/index.php?r=lncbasev2/index) was applied in an attempt to confirm he binding of lncRNA to microRNA (miRNA) and the downstream genes of miRNA were predicted by the databases including TargetScan (Total context +  + score < − 0.05) (http://www.targetscan.org/vert_71/), RAID (Score > 0.5) (http://www.rna-society.org/raid2/index.html), mirDIP (Number of Sources > 3, Score Class: Medium) (http://ophid.utoronto.ca/mirDIP/) and miRWalk (energy < -20, accessibility < 0.01, au > 0.45) (http://mirwalk.umm.uni-heidelberg.de). After the intersection of the predicted downstream genes with the top 500 differentially expressed genes in TCGA, key downstream genes were obtained through the construction of a protein–protein interaction (PPI) network based on String (https://string-db.org) and calculating core degree of them via Cytoscape (https://cytoscape.org) while downstream pathways were analyzed by the co-expression analysis based on MEM (https://biit.cs.ut.ee/mem/index.cgi).

### Tissue sample collection

GC tissues and adjacent normal tissues (over 5 cm away from the tumors) were excised from 64 male patients and 51 female patients, aged between 16 and 76 (mean age: 52.62 ± 13.32, median age: 55). All patients underwent surgery at The People’s Hospital of Kaizhou District from 2015-03-02 to 2017-03-02. After the tissues had been collected, they were immediately stored in − 80 °C liquid nitrogen. All patients had yet to receive any treatment with chemotherapy or radiotherapy prior to surgery, and had their diagnosis confirmed via a pathology test. According to the pathological staging criteria (7th edition) of the *Union for International Cancer Control*, 18 patients were at stage I, 32 in stage II, 39 at stage III, and 26 at stage IV; 44 were poorly differentiated, 33 were moderately differentiated, and 38 were well-differentiated; 61 cases had lymph node metastasis.

### Cell culture, grouping, and transfection

Human GC cell line SGC-7901 (American Type Culture Collection, ATCC, Manassas, VA, USA) was maintained in the Roswell Park Memorial Institute (RPMI)-1640 (South Logan, UT, USA) with 10% fetal bovine serum (Tianhang Biotechnology Co., Ltd., Zhejiang, China) and 100 units/ml penicillin/streptomycin in an incubator with 5% CO_2_ at 37 °C. The medium was renewed every 24—48 h. The cells exhibiting logarithmic growth were subsequently detached using 0.25% trypsin and transfected with CCL21 (150 μg·L^−1^ CCL21, ZSGB-Bio, Beijing, China), oe-MALAT1-1, si-MALAT1-1, si-MALAT1-2, or si-SRSF1 (the above plasmids designed, synthesized and constructed by Shanghai GenePharma Co. Ltd., Shanghai, China) in accordance with the instructions of the Lipofectamine^TM^2000 (Invitrogen, Carlsbad, CA, USA).

### Cell morphology

A phase-contrast microscope (Olympus imt-413, Olympus Optical Co, Ltd., Tokyo, Japan) was used to observe and photograph the morphology of the SGC-7901 cells.

### Scratch test

Following 48 h of transfection, the GC cells were confirmed to have reached 90% confluence. Straight lines were drawn on the bottom of the culture plate with a Marker pen and a sterilized 200 µL pipette tip was used to create a wound along the marked line. The cells were added into a serum-free medium after which the wound was measured and recorded under an optical microscope at 0 and 48 h, respectively, to assess the wound healing status.

### Transwell assay

Next, to identify cell migration, the resuspended cells (3 × 10^4^/mL) with the Opti-MEMI (Invitrogen) containing 10 g/L bovine serum albumin were added to the apical side of a 24-well Transwell chamber (8 μm aperture, Corning, USA), while 600 μL 10% RPMI-1640 medium was added to the basolateral side. After 48 h, the chamber was fixed with 4% paraformaldehyde for 30 min and processed by 0.2% Triton X-100 (Sigma-Aldrich Chemical Company, St Louis, MO, USA) for 15 min, followed by the application of gentian violet staining. The migrated cells were counted under an inverted microscope with five randomly selected fields. The cell invasion assay was performed by coating the chamber with 50 μL Matrigel (Sigma) prior to the experiment with the subsequent steps performed in an identical manner as the aforementioned procedures.

### Reverse transcription quantitative polymerase chain reaction (RT-qPCR)

Total RNA was extracted from GC tissues using a RNA extraction kit (Invitrogen) and then reversely transcribed into cDNA based on the instructions of the PrimeScript RT kit. MALAT1, SRSF1, CCL21, miR-202-3p, glyceraldehyde-3-phosphate dehydrogenase (GAPDH), and U6 primers were designed and synthesized by the Takara (Additional file 1: Table S1). Fluorescence qPCR was performed as per the instructions of the SYBR® Premix Ex Taq™ II kit. GAPDH served as the internal reference for the relative expression of MALAT1, SRSF1, and CCL21 while U6 was regarded as the internal reference for miR-202-3p. Next, in order to identify the expression of mature miRNAs, the TaqMan® microRNA Assay (Applied Biosystems, Foster City, CA) was used based on the manufacturer’s instructions. The quantitative analysis of the change in expression levels was calculated by ABI 7300 real-time PCR machine (Applied Biosystems). The relative transcriptional level of MALAT1 and mRNA were calculated by the 2^−△Ct^ and 2^−ΔΔCt^ methods respectively. RT-qPCR was performed according to the aforementioned protocol for the cell experiment.

### Western blot analysis

Total protein was extracted from SGC-7901 cells with lysis buffer (Shanghai Beyotime Biotechnology Co. Ltd., Shanghai, China) containing a protease inhibitor, separated by 10% sodium dodecyl sulfate–polyacrylamide gel electrophoresis, and electrotransferred onto a polyvinylidene fluoride membrane. The membrane was subsequently blocked using 5% skimmed milk powder at 4 °C overnight, and probed with diluted primary antibodies to SRSF1 (1 µg/mL, ab38017, Abcam, Cambridge, UK), β-catenin (1: 5000, ab32572, Abcam), cyclinD1 (1: 500, ab226977, Abcam), E-Cadherin (1: 50, ab1416, Abcam), Vimentin (1: 10000, 60330-1-Ig, Proteintech, Rosemont, IL, USA), Snail (0.1 µg/mL, ab53519, Abcam), Slug (1: 500, ab27568, Abcam), Twist (0.5 µg/mL, ab50581, Abcam), and GAPDH (1: 500, ab8245, Abcam) at room temperature overnight. Additionally, the membrane was re-probed with secondary antibodies of goat anti-rabbit antibody (1: 5000, ab97080, Abcam) and goat anti-mouse antibody (1: 10000, ab97258, Abcam) for 1 h followed by visualization using enhanced chemiluminescence working fluid (Pierce, Rockford, IL, USA). Finally, ImageJ2x was used during the analysis with GAPDH employed as the internal reference.

### Dual-luciferase reporter gene assay

The HEK-293 T (AT-1592, ATCC) cells were seeded in 24-well plates and cultured for 24 h. The dual-luciferase reporter gene vector for 3′-untranslated region (UTR) of SRSF1, pmiR-RB-Report-SRSF1-3′UTR, was constructed and co-transfected with oe-MALAT1, si-MALAT1, or negative controls (NCs) into HEK-293 T cells. The cells were then lysed at 48 h after transfection, and the dual-Luciferase® Reporter Assay System (E1910, Promega, Madison, WI, USA) was applied to detect luciferase activity. A total of 50 μL Firefly luciferase working fluid and 50 μL Renilla luciferase working fluid were added to each 10 μL cell sample and the ratio of Firefly luciferase activity to Renilla luciferase activity was regarded as the relative luciferase activity.

### Nude mouse xenograft experiment

Specific pathogens-free grade female BALB/C nude mice (age: 4–5 weeks, weight: 19.23 ± 0.92 g) for experimental use were purchased from the Experimental Animal Center of Yangzhou University Medical College and maintained under a controlled constant temperature (20–26 °C) with constant humidity (50–56%). SGC-7901 cells (logarithmic growth) infected with si-MALAT1 or NC lentivirus were selected and detached with mixed liquor containing 0.02% ethylene diamine tetraacetic acid-2Na and 0.25% trypsin followed by 5-min centrifugation for supernatant removal. The cells were resuspended, counted, and adjusted to a density of 5 × 10^5^ /mL. A total of 200 μL cell suspension was subcutaneously injected into the nude mice with the xenografts formed one week later. The major and minor axis of the tumors were measured and recorded every two days. After 6 weeks, the mice were euthanized with the tumors excised and photographed. Half of the tumor tissues were frozen and stored in liquid nitrogen for RNA and protein extraction, while the remaining half was fixed with paraformaldehyde and embedded in paraffin for tissue sectioning. Finally, the tumor volume was calculated followed by plotting of a tumor growth curve.

### RNA pull-down assay

The Magnetic RNA–Protein Pull-Down Kit (Thermo Fisher Scientific) was employed to perform RNA pull-down assay. Biotinylated MALAT1 or miR-202-3p was incubated with streptavidin beads overnight at 4 °C. Cell lysate was added and incubated at 4 °C for 4 h. After washing and eluting, MALAT1 and miR-202-3p were analyzed by RT-qPCR.

### RNA binding protein immunoprecipitation (RIP)

The RIP Kit (Merck Millipore, Billerica, MA, USA) was used to identify the binding of miR-202-3p to MALAT1 and SRSF1. The cells were washed with pre-chilled phosphate buffer saline with the supernatant discarded and lysed with Radio Immunoprecipitation Assay lysis (P0013B, Beyotime) followed by centrifugation at 14,000 rpm for 10 min at 4 °C to collect the supernatant. Half of the supernatant was taken as the input whilst the remaining half was incubated with antibodies for immunoprecipitation. Initially, 50 μL magnetic beads were washed and resuspended in 100 μL RIP Wash Buffer, in which 5 μg antibodies were added. The magnetic bead-antibody complex was subsequently resuspended in 900 μL RIP Wash Buffer followed by the addition of 100 μL cell extract for overnight incubation at 4 °C with the bead-protein complex collected. The RNA of the sample and input was detached using protease K and extracted, after which PCR was performed to detect MALAT1 and miR-202-3p expression. The antibody to Argonaute 2 (AGO2) (ab32381, 1: 1000, Abcam) and antibody to immunoglobulin G (IgG) (1: 100, abl09489) were used for the assay with the latter regarded as the NC.

### Immunohistochemistry (IHC)

Tissue blocks fixed in 4% paraformaldehyde were extracted, embedded in paraffin, and sectioned into 4-μm-thick sections. Then the paraffin-embedded sections were subjected to IHC staining using standard procedures. Sections were incubated with primary antibody followed by incubation with HRP-labeled secondary antibody. The diluted concentrations of antibodies used in IHC were as follows: Snail and Slug (1: 5000, ab224731), Vimentin (1: 200, ab92547) antibodies were from Abcam; SRSF1 (1: 500, PA5-30,220) antibody was from Thermo Fisher. The binding extent of the antibodies was visualized using DAB staining. Tissue sections were re-stained with hematoxylin. The cross-sectional images were taken by LeicaMicrosystems (model: DM2000, CMSGmbH, Wetzlar, Germany). Six fields per section were randomly selected for immunohistochemical scoring by ImagePro Plus 6.0 (Media Cybernetics, Inc., Rockville, MD, USA).

### Terminal deoxynucleotidyl transferase-mediated dUTP nick-end labeling (TUNEL) staining

Tissue sections were dewaxed and rehydrated, and then processed by antigen unmasking using 0.1 M sodium citrate (pH 6.2). Then, each section was incubated with 100 ul DNAse-free proteinase K for 30 min followed by addition of 500 ul diluent and 50 ul of TACS nuclease reaction mix (streptavidin-HRP solution) by capillary action for 30-min incubation. Afterwards, 50 ul of fluorescein-labeled solution and 450 ul of enzyme solution were prepared followed by adding 50 ul of each reagent to the 15 sections for 60-min incubation at 37 °C. Evans blue was used for counterstaining of the sections, which were then a covered with fluorescent mounting medium. Finally, an Olympus BX61 fluorescence microscope was used for examining reactions, and positivity was dichotomously categorized, with the results analyzed by X2 test.

### RNAscope

RNAScope Fluorescent Multiplex (Advanced Cell Diagnostics) was employed according to manufacturer’s instructions accompanied by the following changes. Target Retrieval boiling time was altered to 12 min and incubation by Protease IV at a temperature of 40 °C was altered to 8 min. Sections were mounted with Slowfade Mountant + DAPI (Life Technologies, S36964) and sealed.

### Statistical analysis

SPSS version 21.0 (IBM SPSS Statistics, Chicago, IL, USA) was performed for statistical analysis. Measurement data were expressed as mean ± standard deviation, and two groups of data obeying normal distribution and equal variance in paired design were compared by paired t-test, while data in unpaired design were compared by unpaired t-test. Data between multiple groups were compared using one-way analysis of variance (ANOVA) followed by Tukey's post-hoc test. Data between the groups at different time points were compared using repeated measures ANOVA and Bonferroni’s post hoc test. A value of *p* < 0.05 was considered to indicate a significant difference.

## Results

### CCL21 was a key gene for GC

**Fig. 1:**
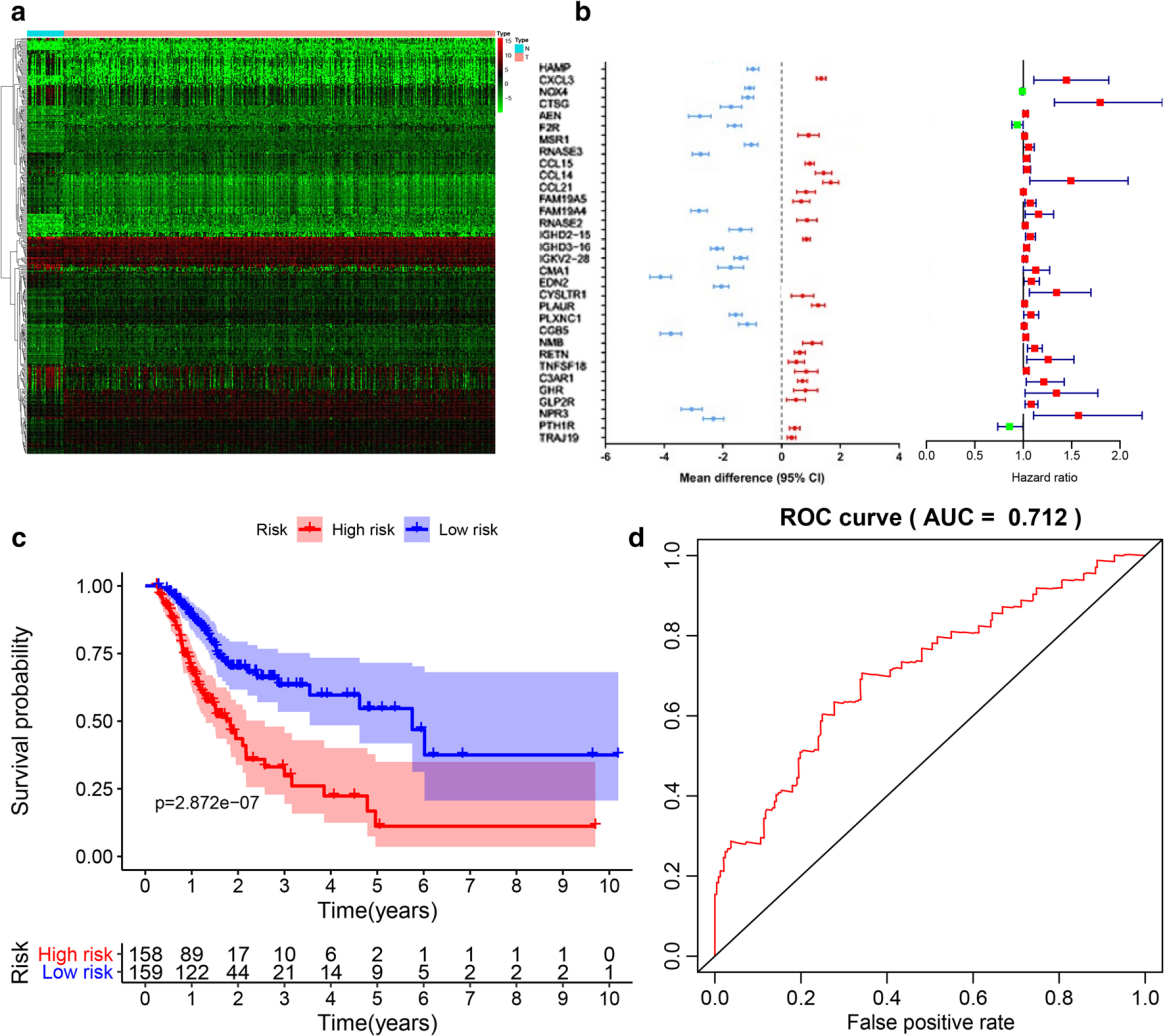
CCL21 was a key gene for GC via prediction using TCGA database. **a** Expression heatmap of 345 significantly differentially expressed immune genes (red: upregulated genes; green: downregulated genes; red/green scale bar relates to the degree of up/downregulated genes relative to normal samples, expressed as logFoldChange) after differential analysis of STAD dataset in TCGA, wherein N represents normal samples and T represents GC samples. **b** 33 out of 345 differentially expressed immune genes were significantly related to the GC prognostic risk (*p* < 0.05, left panel), whose hazard ratio were calculated and plotted at the right panel. **c** The overall survival curve between patients expressing the high-risk and low-risk genes obtained through the multi-factor Cox model. **d** The ROC curve used to verify the survival curve

Through the differential analysis of GC-related immune gene data in the TCGA database, 345 significantly differentially expressed immune genes were identified (Fig. 1a, and Additional file 2: Fig. S1). Based on the clinical data in the TCGA database, single-factor Cox analysis revealed that 33 out of 345 were significantly associated with GC prognostic risk (*p* < 0.05, Fig. 1b). Next, 14 genes (serving as genes for prognostic risk assessment of GC patients) were selected based on the findings of a multi-factor Cox model (Table 1) with the risk scores of clinical samples calculated accordingly. The survival probability curves revealed that the prognosis between patients expressing the high-risk and low-risk genes obtained by our model was significantly different (*p* < 0.001, Fig. 1c). Whilst AUC = 0.712 acquired by the ROC curve provided further evidence confirming that 14 of the genes screened could be used to evaluate the prognosis of GC (Fig. 1d). Moreover, we identified 5 genes with significant differential expression (*p* < 0.01) in the multi-factor Cox model i.e., CXCL3, CCL21, IGHD2-15, PLAUR, and NMB. Combining the results of the differential analysis, we found that CCL21 was the most significantly differentially expressed among these five genes, highlighting its potential as a key gene associated with GC (Table 1).

**Table 1:** Fourteen genes screened out by multi-factor Cox model

GeneSymbol	Multivariate Cox analysis					Difference analysis
	Coef	HR	HR.95L	HR.95H	Cox.P	DEGs.P
CXCL3	− 0.0148	0.9853	0.9747	0.9961	0.0078	8.26E-07
CTSG	− 0.4099	0.6637	0.4703	0.9366	0.0197	3.04E-11
AEN	− 0.0779	0.9251	0.8629	0.9918	0.0284	1.40E-10
RNASE3	0.4521	1.5716	0.9107	2.7123	0.1044	1.21E-03
CCL15	0.033	1.0336	0.9961	1.0724	0.0797	4.48E-06
CCL21	0.0013	1.0013	1.0005	1.002	0.0012	1.02E-08
FAM19A4	0.1378	1.1477	1.0184	1.2935	0.0239	1.94E-05
RNASE2	0.0884	1.0924	1.0135	1.1774	0.0209	3.37E-07
IGHD2-15	0.0904	1.0946	1.044	1.1475	0.0002	2.39E-02
CMA1	0.6172	1.8537	1.1536	2.9786	0.0108	1.26E-12
PLAUR	0.0218	1.022	1.0069	1.0374	0.0043	2.28E-07
NMB	0.0447	1.0458	1.0205	1.0716	0.0003	1.29E-08
TNFSF18	0.18	1.1973	0.9748	1.4705	0.0861	9.23E-05
TRAJ19	− 0.1749	0.8395	0.7126	0.9892	0.0366	2.23E-03

Coef is the coefficient of a gene in the multi-factor COX model; HR is the risk score; HR.95L and HR.95H are the maximum and minimum risk scores, respectively; Cox.P is the significance of a gene in the multi-factor COX model; DEGs.P is the significance of a gene in the differential analysis

### CCL21 promoted the migration and invasiveness as well as EMT of SGC-7901/MGC-803 cells

**Fig. 2:**
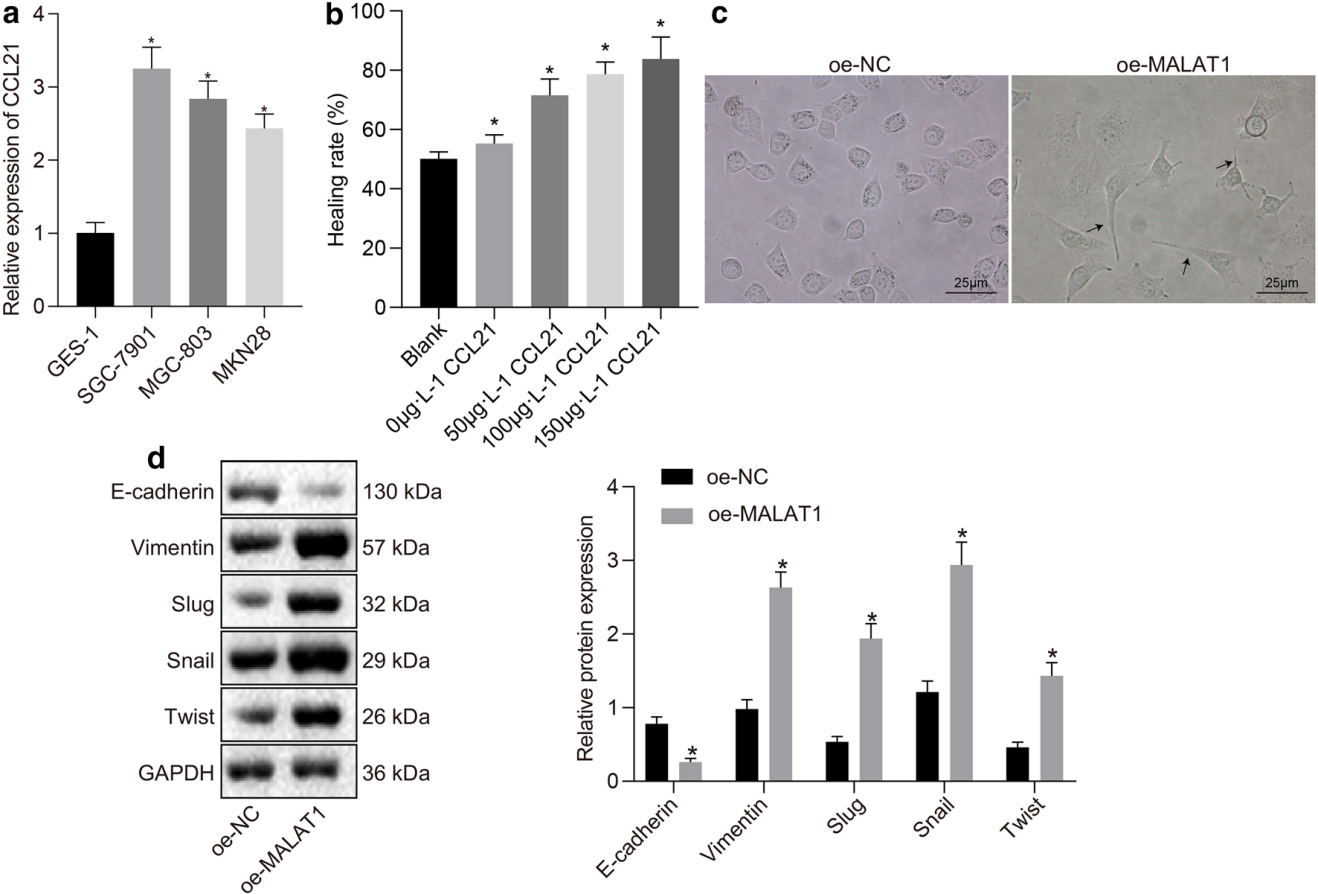
CCL21 promoted migrative and invasive potential as well as EMT of SGC-7901 cells. **a** RT-qPCR determination of the CCL21 expression in GES-1, SGC-7901, MGC-803, and MKN28 cells. **b** Results of scratch wound assay. **c** Representative microscopic images of control SGC-7901 cells and the cells treated with CCL21 (150 μg·L^−1^) for 48 h (the arrow points to pseudopodium formation) (× 400). **d** Western blot analysis of the expression of E-cadherin, Vimentin, Slug, Snail, and Twist after CCL21 (150 μg·L^−1^) treatment normalized to GAPDH. * *p* < 0.05 compared with GES-1 cells, blank cells or oe-NC-treated cells. The values in the figure were all measurement data, expressed as the mean ± standard deviation. Unpaired *t-*test was used for comparing data of two groups, and one-way ANOVA was applied for comparing data of multiple groups followed by Tukey's post-test. The experiment was repeated for three times independently

Next, to investigate the effect of CCL21 on GC, RT-qPCR was performed, the results of which revealed that the expression of CCL21 in human GC cells SGC-7901, MGC-803, and MKN28 was higher than that in normal gastric epithelial cells GES-1 (*p* < 0.05, Fig. 2a). The SGC-7901 and MGC-803 cells with the highest and second highest expression of CCL21 were used for subsequent experiments. The SGC-7901/MGC-803 cells were subsequently cultured with 0, 50, 100, and 150 μg·L^−1^ CCL21 for 48 h, respectively. Our results indicated that the cells treated with 150 μg·L^−1^ CCL21 exhibited the fastest growth and the scratches in cell plate displayed the quickest healing and most significant narrowing (*p* < 0.05) (Fig. 2b). Based on these findings, 150 μg·L^−1^ was selected as the treatment concentration of CCL21 in this experiment. The results from an optical microscope showed that the control SGC-7901/MGC-803 cells exhibited oval-like cell body after adherent growth and the SGC-7901/MGC-803 cells treated with CCL21 (150 μg·L^−1^) for 48 h showed fusiform cell body, some of which had pseudopodium (Fig. 2c). After CCL21 (150 μg·L^−1^) treatment for 48 h, typical features of EMT were observed that SGC-7901/MGC-803 cells demonstrated low expression of E-cadherin (*p* < 0.05) and high expression of Vimentin, Slug, Snail, and Twist (*p* < 0.05, Fig. 2d). The aforementioned results highlighted that CCL21 enhanced migration, invasiveness, and EMT of SGC-7901/MGC-803 cells.

### CCL21 promoted migration, invasion, and EMT of SGC-7901/MGC-803 cells by upregulating the expression of MALAT1

**Fig. 3:**
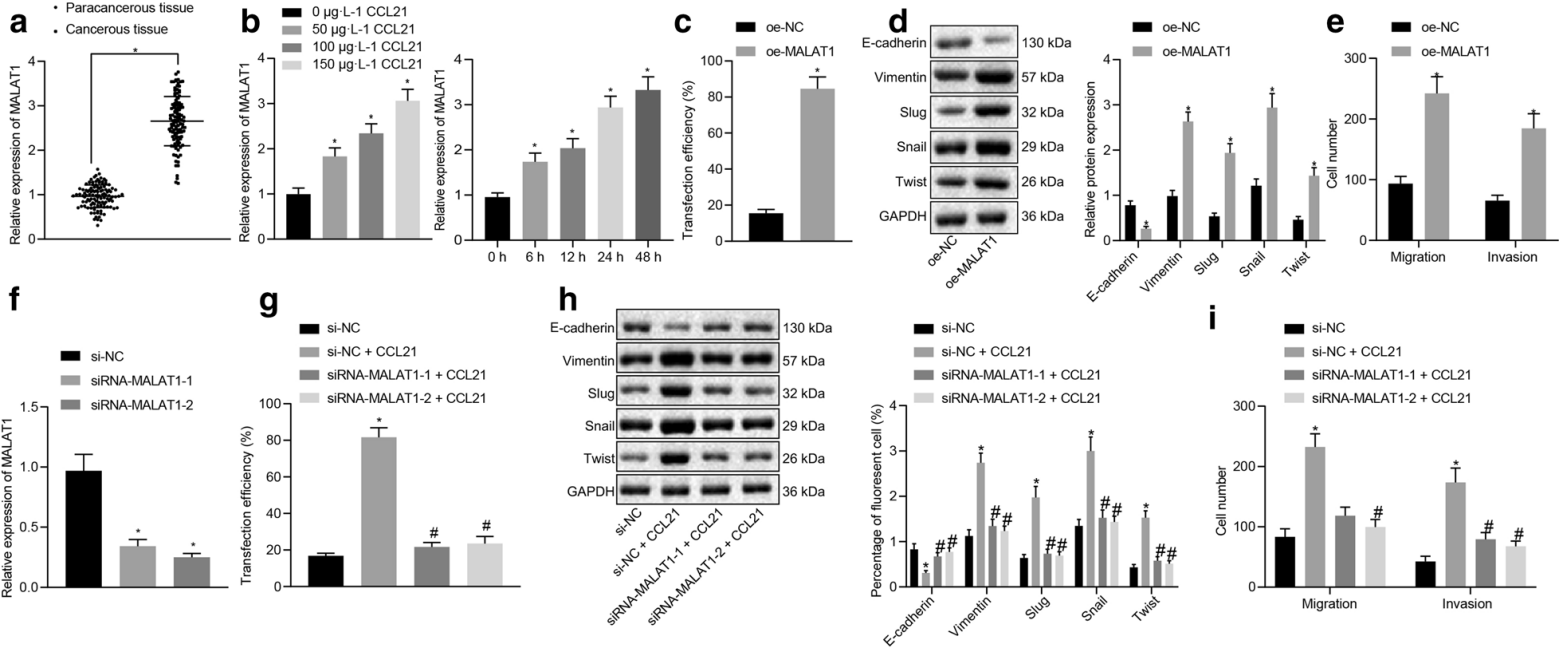
CCL21 increased the expression of MALAT1, thus promoting EMT, migration, and invasiveness of SGC-7901 cells. **a** RT-qPCR determination of the relative expression of MALAT1 in GC tissues and adjacent normal tissues (N = 115). **b** RT-qPCR measurement of the MALAT1 expression in SGC-7901 cells treated with different concentrations of CCL21 for 48 h and with 150 μg·L^−1^ CCL21 for different times. **c** Assessment of the efficiency of transfecting SGC-7901 cells with MALAT1. **d** Western blot analysis of the effect of CCL21 on the EMT-related factors of SGC-7901 cells after oe-MALAT1 treatment normalized to GAPDH. **e** The migration and invasion capacities of SGC-7901 cells after oe-MALAT1 treatment assessed by Transwell assay. **f** RT-qPCR determination of the expression of MALAT1 in SGC-7901 cells after si-MALAT1-1 or si-MALAT1-2 treatment. **g** Assessment of the efficiency of transfecting SGC-7901 cells with CCL21 after oe-CCL21, si-MALAT1-1, or si-MALAT1-2 treatment. **h** Western blot analysis of the expression of E-cadherin, Vimentin, Slug, Snail, and Twist after oe-CCL21, si-MALAT1-1, or si-MALAT1-2 treatment normalized to GAPDH. **i** The number of migratory cells and invaded cells in each group determined by Transwell assay after oe-CCL21, si-MALAT1-1, or si-MALAT1-2 treatment. * *p* < 0.05 compared with treatment of 0 μg·L^−1^ CCL21, oe-NC, or si-NC. # *p* < 0.05 compared with treatment of si-NC + oe-CCL21. Data in the figure were all measurement data, expressed as the mean ± standard deviation. Data between the two groups of GC tissues and adjacent normal tissues were compared by paired *t*-test, data between the other two groups by unpaired *t*-test, and data between multiple groups by one-way ANOVA and Tukey's post hoc test. The experiment was repeated for three times independently

The expression of MALAT1 in GC was measured by the RT-qPCR experiment. Our results (Fig. 3a) indicated that the relative expression of MALAT1 in GC tissues and adjacent normal tissues was 2.57 ± 0.15 and 0.98 ± 0.13, respectively (*p* < 0.001), suggesting that MALAT1 was highly expressed in GC tissues and could be involved in the occurrence and development of GC. The relative expression of MALAT1 in GC tissues was not found to be correlated to age, sex, or tumor size (*p* > 0.05) but significantly associated with pathological stage, differentiation degree, and lymph node metastasis (*p* < 0.001, Table 2). We subsequently set out to evaluate the effect of CCL21 on the MALAT1 expression. Our RT-qPCR findings indicated that CCL21 induced the MALAT1 expression in SGC-7901 cells in a dose- and time-dependent manner (Fig. 3b).

**Table 2:** The relationship between the MALAT1 expression in GC tissues and clinicopathologic features

Indicators	*N* = *115*	MALAT1 expression	*p* value
Age			
≥ 60	42	2.711 ± 0.593	0.401
< 60	73	2.620 ± 0.535	
Gender			
Male	64	2.677 ± 0.541	0.607
Female	51	2.623 ± 0.578	
Tumor size			
≥ 5	47	2.684 ± 0.452	0.631
< 5	68	2.633 ± 0.621	
Pathological stage			
I + II	50	2.313 ± 0.479	< 0.001
III + IV	65	2.915 ± 0.464	
Differentiation degree			
Low	44	3.030 ± 0.510	< 0.001
Medium–high	71	2.420 ± 0.447	

*lncRNA* long noncoding RNA, *MALAT1* metastasis-associated lung adenocarcinoma transcript 1, *GC* gastric cancer

Moreover, RT-qPCR results confirmed the transfection efficiency of MALAT1 in SGC-7901/MGC-803 cells (Fig. 3c), highlighting that MALAT1 was indeed upregulated. Western blot results revealed that E-cadherin was significantly downregulated while the expression of Vimentin, Slug, Snail, and Twist was markedly elevated following transfection with MALAT1 overexpression vector (*p* < 0.05, Fig. 3d). Transwell assay results documented that the number of migrated and invaded cells was markedly increased after the transfection of the MALAT1 overexpression vector (*p* < 0.05, Fig. 3e), suggesting that upregulation of MALAT1 could promote the migration and invasion abilities of the SGC-7901/MGC-803 cells.

Crucially, our RT-qPCR analysis data provided indication that the expression of MALAT1 was markedly diminished in the cells after the transfection of si-MALAT1-1 or si-MALAT1-2 (*p* < 0.05, Fig. 3f), while CCL21 triggered a significant increase in the expression of MALAT1 (*p* < 0.05). However, the aforementioned observation was reversed by transfection of si-MALAT1 (*p* < 0.05, Fig. 3g). Western blot and Transwell experiments indicated that the expression of E-cadherin was downregulated while Vimentin was upregulated after overexpressing CCL21 and the number of migrated and invaded cells were increased (*p* < 0.05). The effect of upregulated CCL21 was reversed following treatment with si-MALAT1-1 or si-MALAT1-2 (*p* < 0.05, Fig. 3h, i). Altogether, knockdown of MALAT1 expression could significantly inhibit the CCL21-induced EMT, migration, and invasiveness of SGC-7901/MGC-803 cells.

### CCL21 increased the MALAT1 expression to promote the tumorigenesis of SGC-7901/MGC-803 cells in vivo

**Fig. 4:**
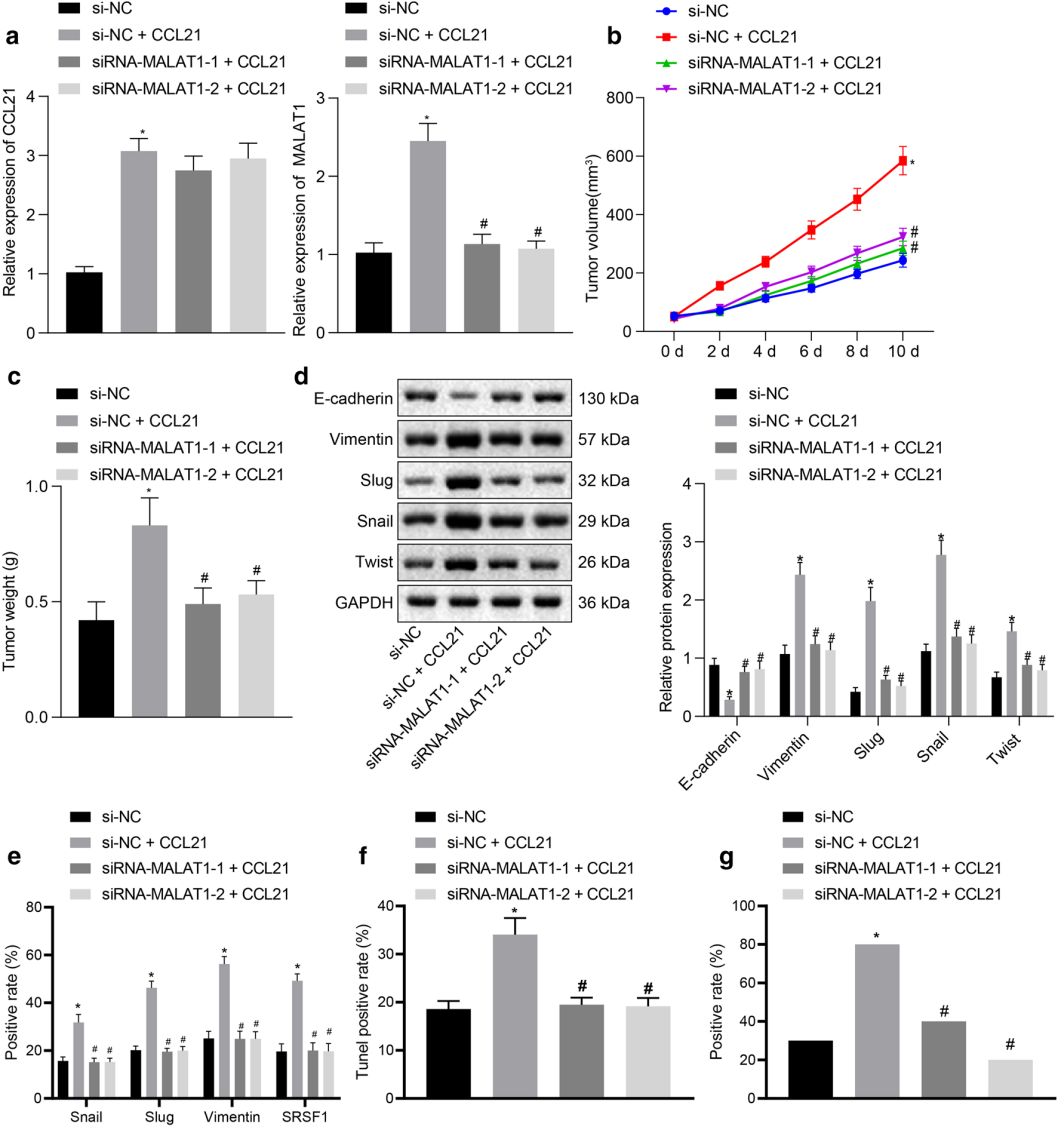
CCL21 elevated the expression of MALAT1 to facilitate the tumorigenesis of SGC-7901 cells *in-vivo*. **a** RT-qPCR determination of the CCL21 and MALAT1 expression after oe-CCL21, si-MALAT1-1, or si-MALAT1-2 treatment. **b** Tumor growth curves after oe-CCL21, si-MALAT1-1, or si-MALAT1-2 treatment. **c** Statistics of tumor weight after oe-CCL21, si-MALAT1-1, or si-MALAT1-2 treatment. **d** Western blot analysis of E-cadherin, Vimentin, Slug, Snail, and Twist after oe-CCL21, si-MALAT1-1, or si-MALAT1-2 treatment normalized to GAPDH. **e** IHC analysis of vimentin, Slug, Snail, and SRSF1 expression in oe-CCL21-, si-MALAT1-1-, or si-MALAT1-2-treated tumor tissues. **f** TUNEL staining analysis of apoptosis in oe-CCL21-, si-MALAT1-1-, or si-MALAT1-2-treated tumor tissues. G, RNAscope analysis of MALAT1 expression changes in oe-CCL21-, si-MALAT1-1-, or si-MALAT1-2-treated tumor tissues. *p < 0.05 compared with treatment of si-NC. #*p* < 0.05 compared with treatment of si-NC + oe-CCL21. Data in the figure were all measurement data, expressed as the mean ± standard deviation. The experiment was repeated for three times independently

SGC-7901/MGC-803 cells stably transfected with si-MALAT1 or NC lentivirus were inoculated subcutaneously into female BALB/C nude mice. RT-qPCR was performed to determine the expression of CCL21 and MALAT1 in tissues (*p* < 0.05, Fig. 4a). On the 16th day after the inoculation, the tumor formation rate of the two groups of cells exhibited a significant difference in relation to tumor volume which was notably reduced after si-MALAT1 treatment (*p* < 0.05). On the 28th day post inoculation, the nude mice were euthanized and tumor growth curves were constructed. Our data indicated that inhibition of MALAT1 expression led to a marked reduction in the rate of tumor formation and the size of the formed tumors. Whereas overexpression of CCL21 triggered an increase in tumor size, while under-expressed MALAT1-1 or MALAT1-2 diminished the tumor volume (*p* < 0.05, Fig. 4b, c).

Western blot analysis was employed to determine the expression of E-cadherin, Vimentin, Slug, Snail, and Twist in tumor tissues. Our results revealed that upregulated CCL21 reduced the expression of E-cadherin while enhanced the expression of Vimentin, Slug, Snail, and Twist in the tissues (*p* < 0.05). Decreased MALAT1 was found to have a reversal effect on the aforementioned findings exerted by CCL21, which was accompanied by elevated expression of E-cadherin and decreased Vimentin, Slug, Snail, and Twist expression in the tissues (*p* < 0.05, Fig. 4d). The results of IHC similarly showed that CCL21 could up-regulate the expression of Snail, Slug, vimentin, and SRSF1 (*p* < 0.05, Fig. 4e). TUNEL staining results indicated that CCL21 inhibited the apoptosis of cancer cells (*p* < 0.05, Fig. 4f). RNAscope analysis indicated that CCL21 up-regulated the expression of MALAT1 (*p* < 0.05, Fig. 4g).

### MALAT1 inhibited the miR-202-3p expression to upregulate SRSF1

**Fig. 5:**
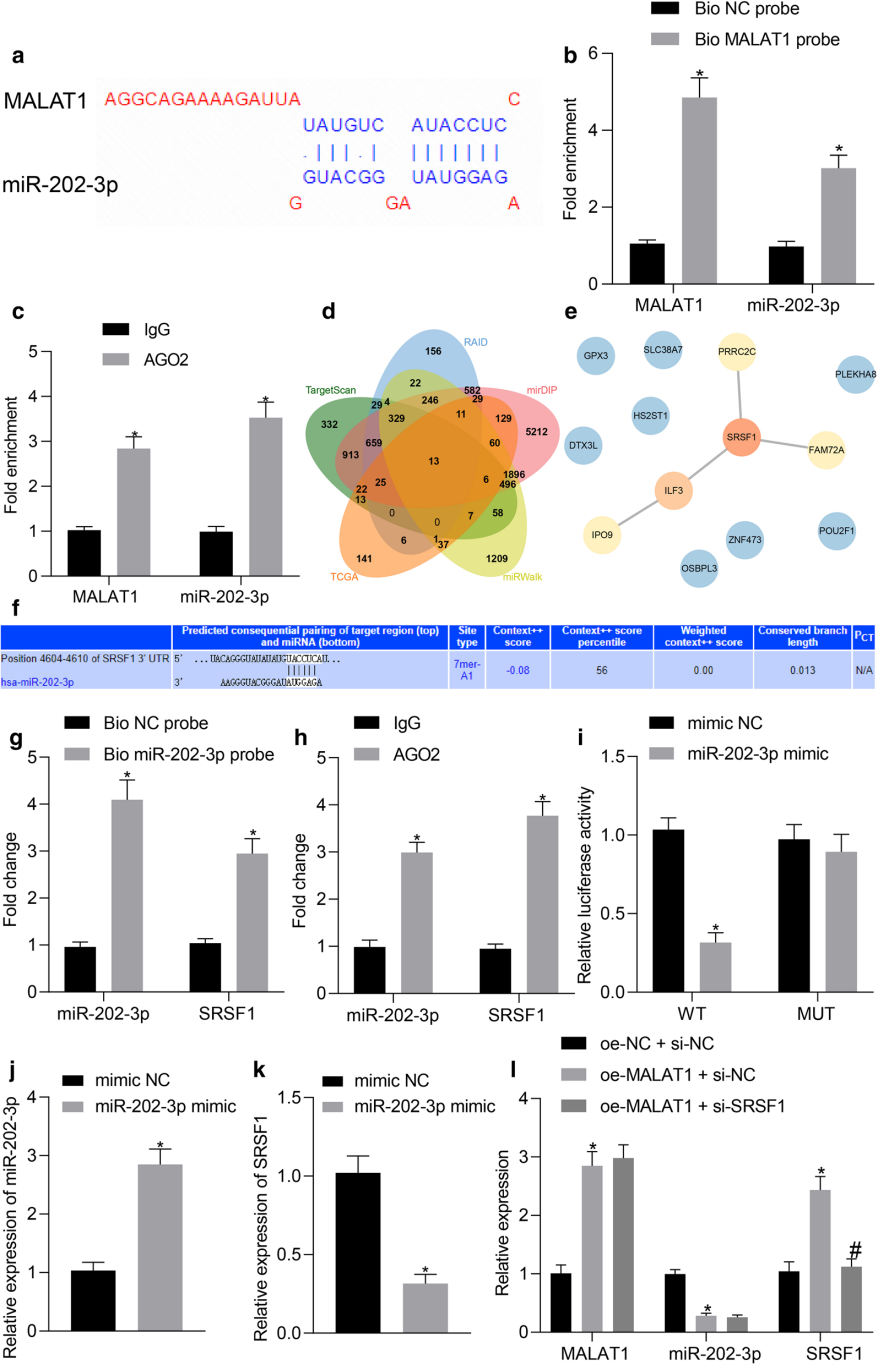
MALAT1 reduced the miR-202-3p expression to upregulate SRSF1. **a** The binding site between MALAT1 and miR-202-3p predicted by LncBase. **b** The interaction between MALAT1 and miR-202-3p verified by RNA pull-down experiment. **c** RIP detection of the binding of MALAT1 to miR-202-3p. **d** Venn diagram displaying the intersection of the downstream genes of miR-202-3p predicted by TargetScan, RAID, mirDIP, and miRWalk and the top 500 differentially expressed genes in the STAD dataset of TCGA. **e** PPI network of the obtained genes through intersection constructed by String, wherein red represents high core degree and blue represents low core degree of the genes, of which the reddest gene is the most core. **f** TargetScan prediction of the binding site between miR-202-3p and SRSF1. **g** RNA pull-down experiment verifying the interaction between miR-202-3p and SRSF1. **h** RIP detection of the binding of miR-202-3p to SRSF1. **i** Luciferase assay detecting luciferase activity in each group after transfection. **j** RT-qPCR determination of the miR-202-3p expression in each group. **k** RT-qPCR determination of the SRSF1 expression in each group. **l** RT-qPCR determination of the MALAT1, miR-202-3p, and SRSF1 expression in each group. *p < 0.05 compared with treatment of Bio NC probe, IgG, mimic NC, and oe-NC + si-NC. #*p* < 0.05 compared with treatment of oe-MALAT1 + si-NC. The values in the figure were all measurement data, expressed as the mean ± standard deviation. Data between the two groups were compared by unpaired *t*-test and data between multiple groups by one-way ANOVA and Tukey's post hoc test. The experiment was repeated for three times independently

Existing literature has previously suggested that MALAT1 targets the miR-202 [18] in GC and that miR-202-3p is lowly expressed in GC [19]. Hence, we set out to further verify the targeting relationship between MALAT1 and miR-202-3p through the LncBase analysis (Fig. 5a). In light of this, the interaction between MALAT1 and miR-202-3p was subsequently confirmed via a RNA pull-down experiment coupled with a RIP experiment. The RNA pull-down experiment results revealed that both MALAT1 and miR-202-3p were notably enriched with biotin-labeled MALAT1 pull-down complex, indicating that MALAT1 could directly bind to miR-202-3p (Fig. 5b). In the RIP experiment, MALAT1 and miR-202-3p coexisted in the product precipitated by the argonaute RISC catalytic component 2 (AGO2) antibody (*p* < 0.05, Fig. 5c).

TargetScan, RAID, mirDIP, and miRWalk were explored to predict the downstream genes of miR-202-3p with 2906, 2112, 10628, and 4395 genes obtained. Through systematic comparisons of the top 500 differentially expressed genes of the STAD dataset in TCGA, a Venn diagram was constructed which revealed the intersection of 13 key genes (Fig. 5d) with a PPI network of these 13 genes established by String. Our results revealed that SRSF1 exhibited the highest core degree among them (Fig. 5e), suggesting that SRSF1 could be the key downstream gene of miR-202-3p and its binding site was predicted by TargetScan (Fig. 5f). RNA pull-down and RIP experiments were conducted to verify the relationship between miR-202-3p and SRSF1. The RNA pull-down experiment results revealed that both miR-202-3p and SRSF1 were significantly enriched with biotin-labeled miR-202-3p pull-down conjugate, indicating that miR-202-3p could directly bind to the SRSF1 (Fig. 5g). In the RIP experiment, miR-202-3p and SRSF1 co-existed in the product precipitated by the AGO2 antibody (*p* < 0.05, Fig. 5h). After the miR-202-3p mimic and reporter gene vector were co-transfected into HEK293T cells for 24 h, after which a luciferase assay was performed with the results indicating that the relative luciferase activity was markedly reduced following co-transfection with miR-202-3p mimic and SRSF1 3′UTR-wild type (*p* < 0.05). No significant difference in terms of luciferase activity after co-transfection with miR-202-3p mimic and SRSF1 3′UTR-mutant was detected (Fig. 5i), implying that miR-202-3p mimic could directly bind to the corresponding site in SRSF1 3′UTR in GC cells. The expression of miR-202-3p and SRSF1 in GC cells assessed by the RT-qPCR clarified that overexpressed miR-202-3p markedly reduced the expression of SRSF1 (*p* < 0.05, Fig. 5j, k). Moreover, upregulation of MALAT1 significantly increased the SRSF1 expression but deceased miR-202-3p expression, while low expression of SRSF1 exerted no significant effect on MALAT1 and miR-202-3p (*p* < 0.05, Fig. 5l). The above-mentioned results supported the notion that MALAT1 could increase SRSF1 expression by reducing miR-202-3p.

### MALAT1 promoted EMT of SGC-7901/MGC-803 cells by activating the mTOR signaling pathway through increasing SRSF1 expression

**Fig. 6:**
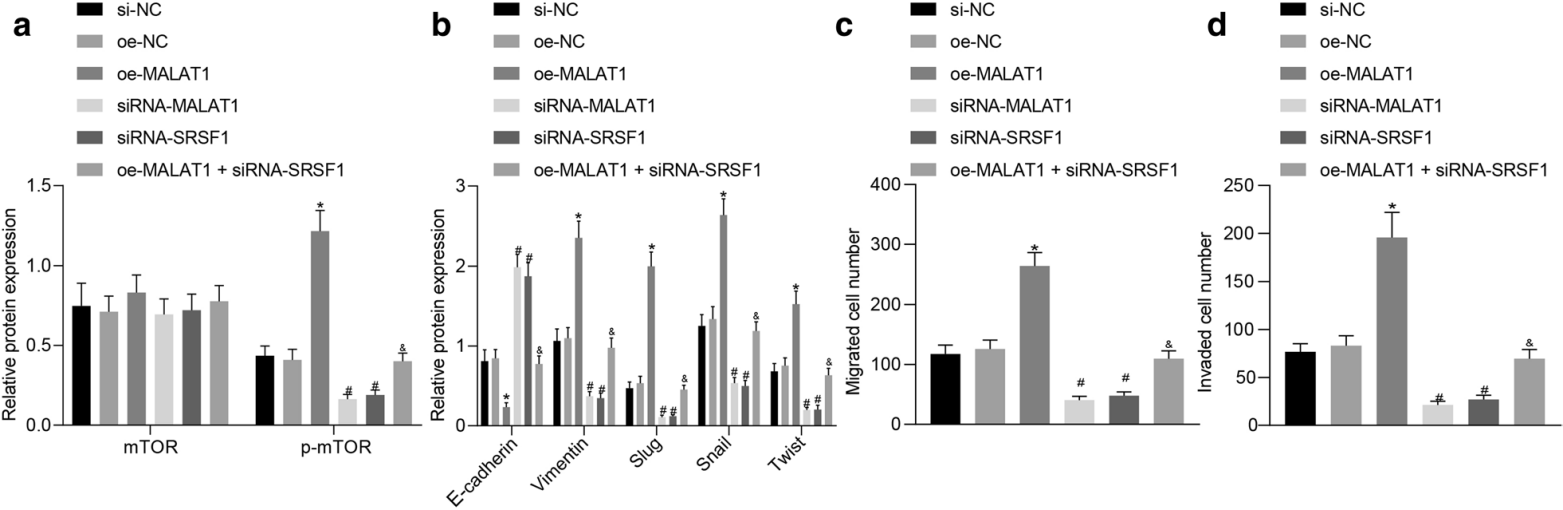
MALAT1 activated the mTOR signaling pathway and EMT of SGC-7901 cells by increasing SRSF1 expression. **a** Western blot analyses of the extent of mTOR phosphorylation after oe-MALAT1, si-MALAT1, or si-SRSF1 treatment normalized to GAPDH. **b** Western blot analysis of the expression of E-cadherin, Vimentin, Slug, Snail, and Twist after oe-MALAT1, si-MALAT1, or si-SRSF1 treatment normalized to GAPDH. **c** Transwell assay displaying the migration of SGC-7901 cells after oe-MALAT1, si-MALAT1, or si-SRSF1 treatment. **d** Transwell assay displaying the invasion of SGC-7901 cells after oe-MALAT1, si-MALAT1, or si-SRSF1 treatment. * *p* < 0.05 compared with treatment of si-NC. # *p* < 0.05 compared with treatment of oe-NC. & *p* < 0.05 compared with treatment of oe-MALAT1. The values in the figure were all measurement data, expressed as the mean ± standard deviation. Data between multiple groups were analyzed by one-way ANOVA and Tukey's post hoc test. The experiment was repeated for three times independently

In the SGC-7901/MGC-803 cell line, RT-qPCR and Western blot were performed to detect the effect of MALAT1-induced SRSF1 upregulation on the activity of mTOR signaling pathway. The results obtained revealed no significant difference in relation to the total protein expression of mTOR in each group; overexpressed MALAT1 led to an increase the extent of mTOR phosphorylation. In addition, inhibition of MALAT1 or SRSF1 significantly inhibited the extent of mTOR phosphorylation; overexpressing MALAT1 while inhibiting SRSF1 led to no notable changes in terms of the extent of mTOR phosphorylation (Fig. 6a). The above-mentioned results provided evidence indicating that MALAT1 triggered an increase in the expression of SRSF1 to activate the mTOR signaling pathway. After 24 h of transfection, Western blot revealed that the upregulation of MALAT1 further enhanced the expression of Vimentin, Slug, Snail, and Twist but decreased the expression of E-cadherin (*p* < 0.05). However, these changes were reversed by si-SRSF1 (*p* < 0.05, Fig. 6b. Thus, the aforementioned results supported that low expression of MALAT1 and SRSF1 could upregulate E-cadherin expression but downregulate the expression of Vimentin, Slug, Snail, and Twist.

Transwell assay was conducted to determine the number of migrated and invaded SGC-7901/MGC-803 cells in each field of vision (*p* < 0.05, Fig. 6c, d), the result of which revealed that overexpressed MALAT1 increased the number of migrated and invaded cells. However, silencing of MALAT1 or SRSF1 led to a significant decrease in the number of migrated and invaded cells (*p* < 0.05). Whilst the promotive effect of MALAT1 on the number of migrated and invaded cells were rescued by downregulating SRSF1 (*p* < 0.05). Collectively, MALAT1 and SRSF1 could promote the migrative and invasive potentials of SGC-7901/MGC-803 cells.

### MALAT1 promoted the tumorigenesis of SGC-7901/MGC-803 cells in vivo by enhancing SRSF1 expression

**Fig. 7:**
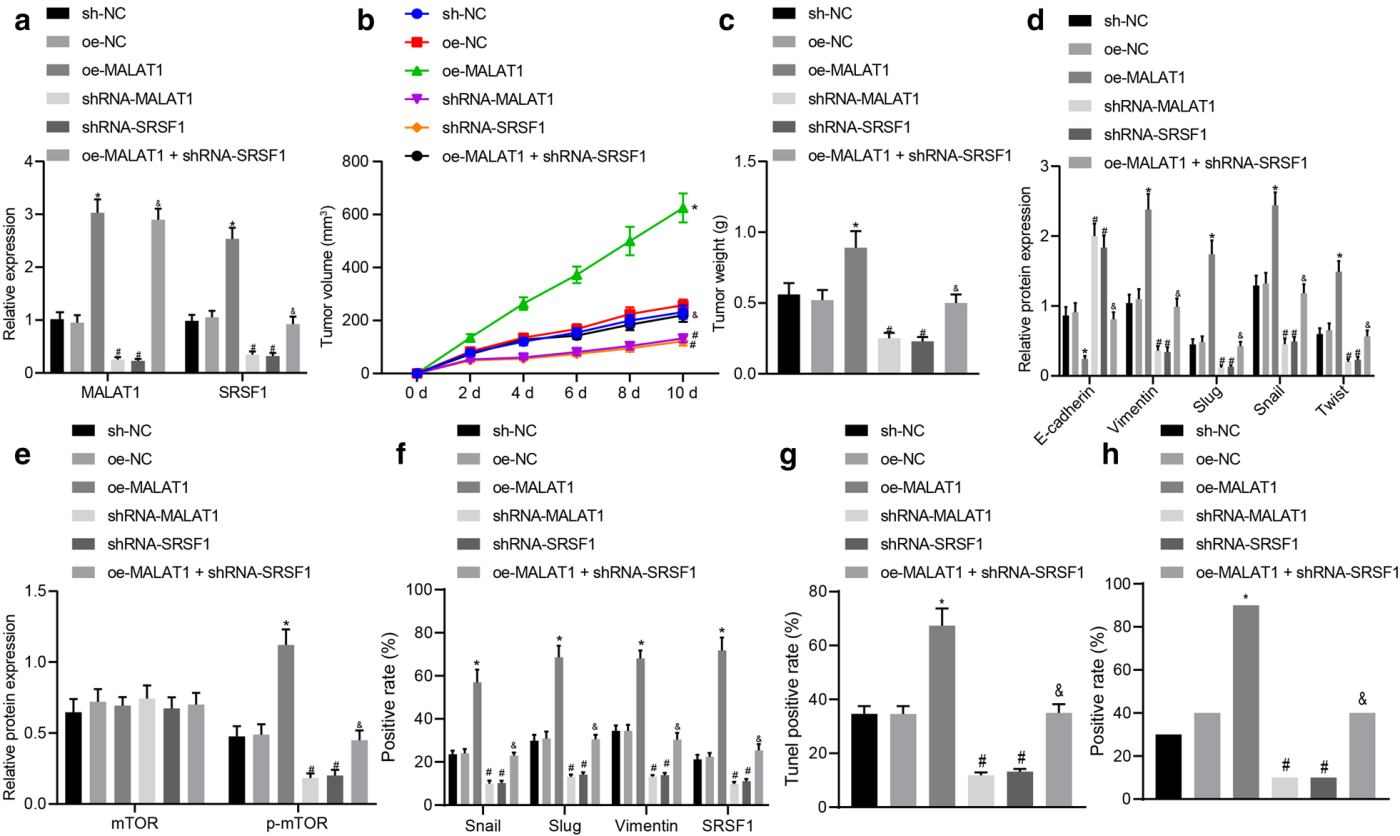
MALAT1 facilitated the tumorigenesis of SGC-7901 cells in vivo by upregulating the expression of SRSF1. **a** RT-qPCR determination of the expression of MALAT1 and SRSF1 after oe-MALAT1, sh-MALAT1, or sh-SRSF1 treatment. **b** Tumor growth curves after oe-MALAT1, sh-MALAT1, or sh-SRSF1 treatment. **c** Tumor weight after oe-MALAT1, sh-MALAT1, or sh-SRSF1 treatment. **d** Western blot analysis of the expression of EMT-related factors after oe-MALAT1, sh-MALAT1, or sh-SRSF1 treatment normalized to GAPDH. **e** Western blot analysis of the mTOR expression and the extent of mTOR phosphorylation after oe-MALAT1, sh-MALAT1, or sh-SRSF1 treatment normalized to GAPDH. **f** IHC analysis of vimentin, Slug, Snail, and SRSF1 expression in oe-MALAT1-, sh-MALAT1-, or sh-SRSF1-treated tumor tissues. **g** TUNEL staining analysis of apoptosis in oe-MALAT1-, sh-MALAT1-, or sh-SRSF1-treated tumor tissues. **h** RNAscope analysis of changes in MALAT1 expression in MALAT1-, sh-MALAT1-, or sh-SRSF1-treated tumor tissues. The values in the figure were all measurement data and expressed as the mean ± standard deviation. Data among multiple groups were analyzed by one-way ANOVA and Tukey's post hoc test, and data among groups at different times were analyzed by repeated-measures ANOVA followed by Bonferroni’s post hoc test, n = 10. The experiment was repeated for three times independently. *p < 0.05 compared with the sh-NC group; #*p* < 0.05 compared with the oe-NC group; & *p* < 0.05 compared with the oe-MALAT1 group

SGC-7901/MGC-803 cells transfected with oe-MALAT1, sh-MALAT1, or sh-SRSF1 were inoculated subcutaneously in female BALB/C nude mice. RT-qPCR was performed to determine the expression of MALAT1 and SRSF1 in tissues (*p* < 0.05, Fig. 7a). On the 16th day post inoculation, our results demonstrated that depletion of MALAT1 or SRSF1 further reduced the tumor volume while upregulation of MALAT1 markedly increased the tumor size (*p* < 0.05). On the 28th day, the nude mice were euthanized after which a tumor growth curve was drawn. Our results suggested that inhibiting the expression of MALAT1 or SRSF1 significantly slowed tumor formation in addition to reducing the size of the formed tumors (*p* < 0.05, Fig. 7b, c).

Additionally, Western blot analysis demonstrated that overexpression of MALAT1 downregulated the E-cadherin expression but upregulated the expression of Vimentin, Slug, Snail, and Twist, while depletion of MALAT1 or SRSF1 resulted in a contrasting trend of results. The regulatory effect of MALAT1 on the above factors was reversed by sh-SRSF1 (*p* < 0.05, Fig. 7d). Furthermore, the Western blot analysis results further demonstrated that the up-regulation of MALAT1 led to a marked increase in the extent of mTOR phosphorylation, while reduction of MALAT1 or SRSF1 resulted in a decrease in the extent of mTOR phosphorylation. The promotive effect of MALAT1 in relation to the extent of mTOR phosphorylation was reversed by under-expressing SRSF1 (*p* < 0.05, Fig. 7e). Thus, we concluded that MALAT1 could promote the tumorigenesis of SGC-7901/MGC-803 cells *in-vivo* by increasing the expression of SRSF1. The results of IHC also showed that overexpression of MALAT1 could up-regulate the expression of Snail, Slug, vimentin, and SRSF1, and silencing MALAT1 or SRSF1 could down-regulate the expression of Snail, Slug, vimentin, and SRSF1 (*p* < 0.05, Fig. 7f). TUNEL staining results indicated that overexpression of MALAT1 inhibited the apoptosis of cells, and silencing MALAT1 or SRSF1 promoted the apoptosis of cells (*p* < 0.05, Fig. 7g). RNAscope analysis revealed that overexpression of MALAT1 elevated the expression of MALAT1, and silencing MALAT1 or SRSF1 inhibited MALAT1 expression (*p* < 0.05, Fig. 7h).

## Discussion

**Fig. 8:**
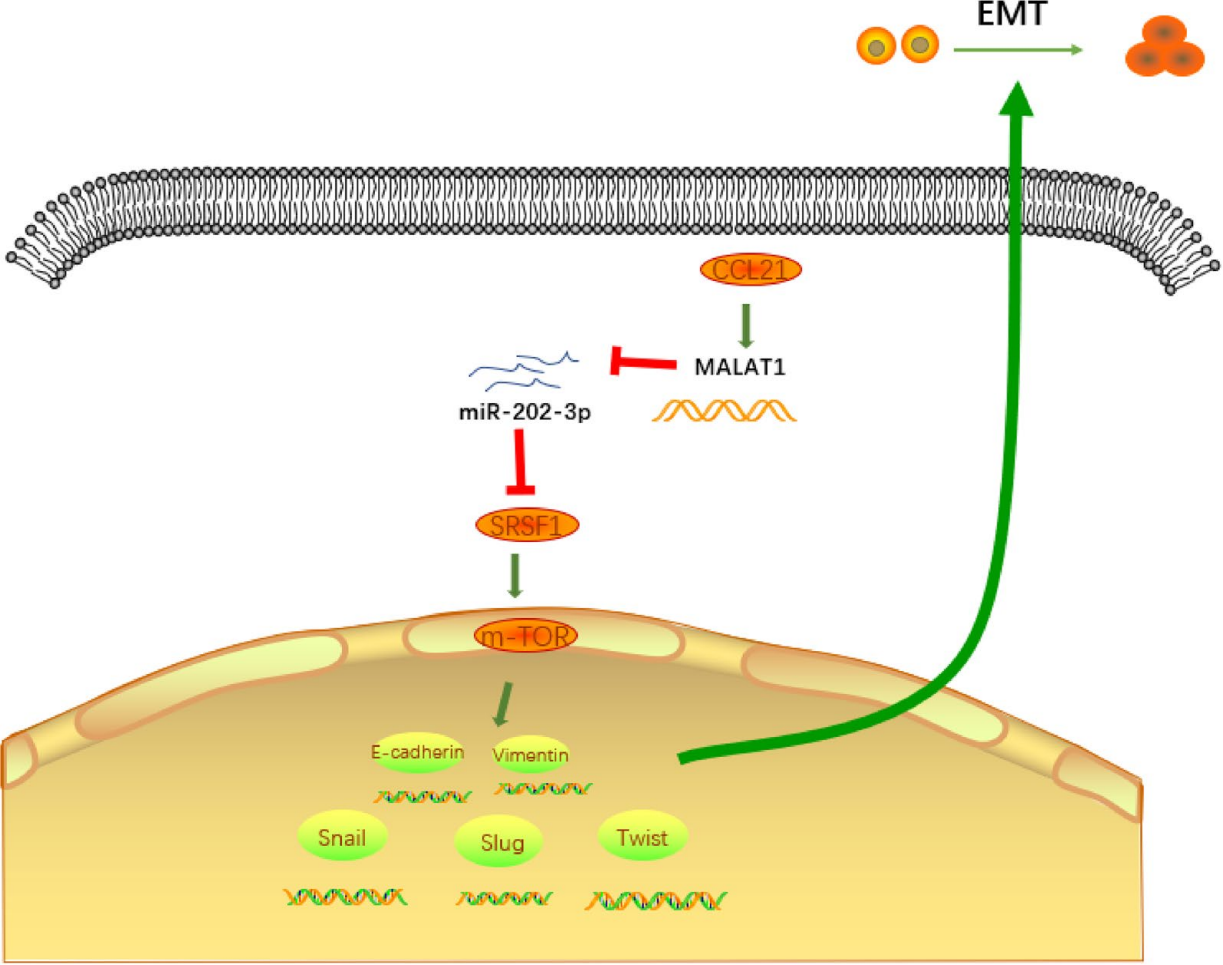
The gene CCL21 induces the expression of MALAT1, and MALAT1 further increases the SRSF1 expression to activate the mTOR pathway, thereby promoting the migration as well as EMT of GC cells and ultimately contributing to the development of GC

The heavy burden of GC remains a problem particularly in Asia, with studies indicating a five-year survival rate for only 5% of patients with GC [2, 20]. More recently, novel immunological therapies have been considered crucial tools capable of treating patients with GC at different stages in addition to more traditional therapies such as surgery, radiotherapy, chemotherapy [21]. In order to identify novel prognostic immune markers for GC, a deeper understanding of the relationship between GC and the host immune microenvironment as well as the means by which immune escape mechanisms take place in cancer initiation and development is required [22]. Existing literature has emphasized the chemokine CCL21 as a therapeutic biomarker for solid human cancers while highlighting its upregulation in GC [8, 9], suggesting its significance as a gene of GC. Thus, during the current study, we aimed to elucidate the role of CCL21 in the development of GC through a series of *in-vitro* and *in-vivo* assays. Our results demonstrated that CCL21 induced the MALAT1-regulated SRSF1 expression to activate the mTOR pathway, ultimately promoting the migration, and EMT of GC cells to facilitate GC growth (Fig. 8).

Our initial bioinformatics analysis data revealed CCL21 as a key gene for GC and further determined that high expression of CCL21 contributed to the malignant phenotypes and EMT of the SGC-7901/MGC-803 cells. Partially consistent with our findings, the secretion of CCL21 has been previously reported in a variety of cancers including GC. Moreover, the ability of CCL21 to transform the immunogenic host immune response to tolerogenic response has been reported, which ultimately stimulates tumor progression [23]. In addition, CCL21 and CXC chemokine receptor 7 have been illustrated as important regulators in the EMT process in human chondrosarcoma [24]. Consistently, our results demonstrated that CCL21 promoted the invasion, migration, and EMT of GC cells *in-vitro* as well as it also promoted the tumorigenesis of SGC-7901/MGC-803 cells *in-vivo* by the upregulation of MALAT1 expression. Accordingly, Hong et al*.* concluded that CCL21 can not only regulate cell migration but also enhance the expression of MALAT1 and activate the mTOR pathway in mycosis fungoides cells [10]. In line with our study observations, Deng et al*.* previously reported that depletion of MALAT1 inhibits tumor metastasis in mouse GC tissues [11]. The results of previous studies have shown that CCL21 is closely related to the occurrence and development of GC, and CCL21 regulates the apoptosis of T lymphocytes [25, 26], which also partially agrees with our findings.

Furthermore, our results revealed significantly reduced expression of miR-202-3p in GC while expression of MALAT1 was enhanced and further targeted miR-202 in GC cells to increase the expression of SRSF1. Accordingly, the interaction between miR-202 and MALAT1 has been previous reported in studies demonstrating that MALAT1 promoted the proliferative potential of GC cells through downregulation of the miR-202 expression [18]. Moreover, as a tumor inhibitor of GC, miR-202-3p has been shown to be attenuated in GC tissues [19]. Our Targetscan prediction results revealed that miR-202-3p targeted SRSF1, which was further confirmed by the RNA pull-down and RIP experiments Consistently, SRSF1 has reported being activated by MALAT1 in colorectal cancer cells [27]. Evidence has been previously presented highlighting the presence of overexpressed SRSF1 in GC cells [14]. Thus, the aforementioned studies lend support to the notion that MALAT1 contributes to GC cell functions in the form of regulating the expression of SRSF1 and miR-202.

Furthermore, our results demonstrated that MALAT1 and SRSF1 inhibited the expression of E-cadherin while triggering an increase in the expression of Vimentin, Slug, Snail, and Twist, suggesting that MALAT1 enhances the expression of SRSF1 to activate mTOR pathway and promote the EMT of GC cells. The role of SRSF1 as an oncogene for HCC has been proposed in previous research by means of activating the mTORC1 pathway [12]. Activation of the mTOR pathway has been reported to induce GC cell growth [15]. Nevertheless, activation of EMT by SRSF1 has been demonstrated to result in cell locomotion [28]. However, the mTOR pathway acts as a pivotal regulator in the EMT process [16], suggesting the interaction among SRSF1, mTOR, and the EMT.

We further attempted to confirm the regulatory role of MALAT1 and SRSF1 in GC development via an *in-vivo* experiment with our results indicating that MALAT1 and SRSF1 facilitated the GC formation in mice. Collectively, our study revealed that CCL21 induces MALAT1 expression to upregulate the expression of SRSF1. SRSF1 further activates the mTOR pathway, thus contributing to the migration, and EMT of GC cells, promoting the occurrence and development of GC.

## Conclusions

Taken together, the key observations of our study provide evidence suggesting the CCL21/MALAT1/SRSF1/mTOR axis as a critical component in the initiation of GC, thereby highlighting CCL21-based immunotherapy as a promising therapy for GC-related pathogenic symptoms. However, further details are required to further elucidate the role of CCL21 in the development of GC by the MALAT1-regulated SRSF1/mTOR axis to develop the novel effective drugs targeting the specific proteins and pathways to treat GC.

## Supplementary Information

**Additional file 1: Table S1.** Primer sequences for RT-qPCR.

**Additional file 2: Fig. S1.** The expression of CXCL3, CCL21, IGHD22-15, PLAUR, and MMB in GC tumor tissues (n = 375) and normal tissues (n = 32). A, the expression of CXCL3; B, the expression of CCL21; C, the expression of IGHD22-15; D, the expression of PLAUR; E, the expression of MMB (JPG 414 KB)

## Data Availability

The datasets generated and/or analysed during the current study are available from the corresponding author on reasonable request.
