# Supplementary material for: CCL21 activation of the MALAT1/SRSF1/mTOR axis underpins the development of gastric carcinoma
Source: J Transl Med. 2021 May 17;19:210. doi: 10.1186/s12967-021-02806-5 (PMC8127212; doi:10.1186/s12967-021-02806-5)
Supplement: Supplementary file 1 — Additional file 1: Table S1. Primer sequences for RT-qPCR. [file 12967_2021_2806_MOESM1_ESM.doc]

**Supplementary Table 1** Primer sequences for RT-qPCR

|  | Sequences |
| --- | --- |
| LncRNA-MALAT1 | F: 5'-AAGCAAGGTCTCCCCACAAG-3' |
|  | R: 5'-GGTCTGTGCTAGATCAAAGGCA-3' |
| SRSF1 | F: 5'-GCCCCGCAGGGAACAACGAT-3' |
|  | R: 5'-CGTCTCGCGGGTCCTCGAAC-3' |
| CCL21 | F: 5'-CCTTATCCTGGTTCTGGCCT-3' |
|  | R: 5'-CAGCCTAAGCTTGGTTCCTG-3' |
| miR-202-3p | F: 5'-AGAGGTATAGGGCATGGGAA-3' |
|  | R: 5'-GGAGACCGCCTGGGAATA-3' |
| GAPDH | F: 5'-CCTGCACCACCAACTGCTTA-3' |
|  | R: 5'-GGCCATCCACAGTCTTCTGAG-3' |
| U6 | F: 5'-CTCGCTTCGGCAGCACA-3' |
|  | R: 5'-AACGCTTCACGAATTTGCGT-3' |

Note: RT-qPCR, reverse transcription quantitative polymerase chain reaction; lncRNA, long noncoding RNA; MALAT1, metastasis-associated lung adenocarcinoma transcript 1; SRSF1, serine arginine-rich splicing factor 1; CCL21, cytokine C-C motif chemokine ligand 21; miR, microRNA; GAPDH, glyceraldehyde-3-phosphate dehydrogenase; F, forward; R, reverse.
