# Supplementary figures and images for: CCL21 activation of the MALAT1/SRSF1/mTOR axis underpins the development of gastric carcinoma
Source: J Transl Med. 2021 May 17;19:210. doi: 10.1186/s12967-021-02806-5 (PMC8127212; doi:10.1186/s12967-021-02806-5)

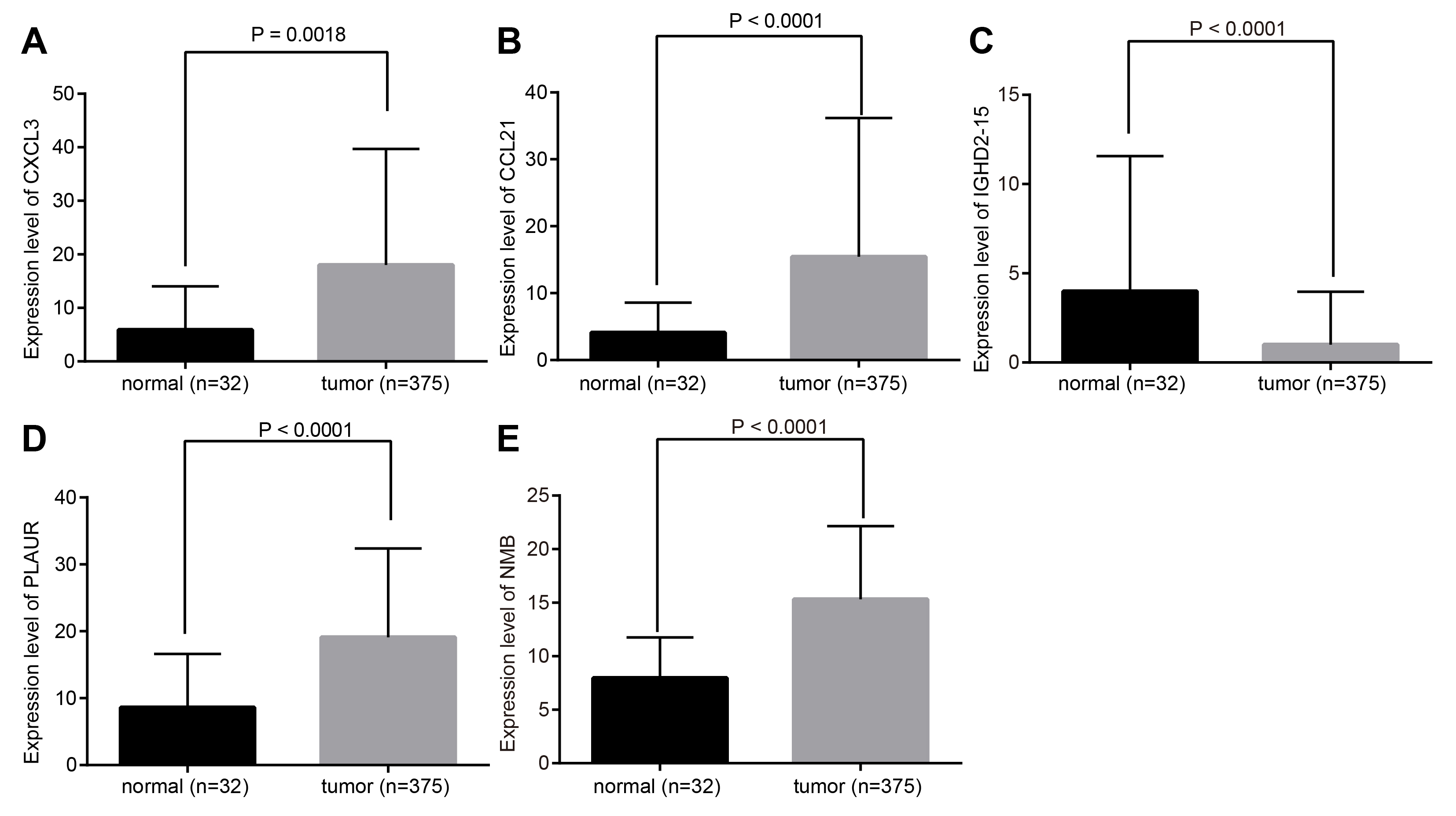

Supplement: Supplementary file 2 — Additional file 2: Fig. S1. The expression of CXCL3, CCL21, IGHD22-15, PLAUR, and MMB in GC tumor tissues (n = 375) and normal tissues (n = 32). A, the expression of CXCL3; B, the expression of CCL21; C, the expression of IGHD22-15; D, the expression of PLAUR; E, the expression of MMB (JPG 414 KB) [file 12967_2021_2806_MOESM2_ESM.jpg]
